# Genome-Wide Identification and Expression Analysis of the NAC Transcription Factor Family in Cassava

**DOI:** 10.1371/journal.pone.0136993

**Published:** 2015-08-28

**Authors:** Wei Hu, Yunxie Wei, Zhiqiang Xia, Yan Yan, Xiaowan Hou, Meiling Zou, Cheng Lu, Wenquan Wang, Ming Peng

**Affiliations:** Key Laboratory of Biology and Genetic Resources of Tropical Crops, Institute of Tropical Bioscience and Biotechnology, Chinese Academy of Tropical Agricultural Sciences, Xueyuan Road 4, Haikou, Hainan, 571101, People’s Republic of China; National Taiwan University, TAIWAN

## Abstract

NAC [no apical meristem (NAM), Arabidopsis transcription activation factor [ATAF1/2] and cup-shaped cotyledon (CUC2)] proteins is one of the largest groups of plant specific transcription factors and plays a crucial role in plant growth, development, and adaption to the environment. Currently, no information is known about the NAC family in cassava. In this study, 96 *NAC* genes (*MeNAC*s) were identified from the cassava genome. Phylogenetic analysis of the NACs from cassava and Arabidopsis showed that MeNAC proteins can be clustered into 16 subgroups. Gene structure analysis found that the number of introns of *MeNAC* genes varied from 0 to 5, with the majority of *MeNAC* genes containing two introns, indicating a small gene structure diversity of cassava *NAC* genes. Conserved motif analysis revealed that all of the identified MeNACs had the conserved NAC domain and/or NAM domain. Global expression analysis suggested that *MeNAC* genes exhibited different expression profiles in different tissues between wild subspecies and cultivated varieties, indicating their involvement in the functional diversity of different accessions. Transcriptome analysis demonstrated that *MeNACs* had a widely transcriptional response to drought stress and that they had differential expression profiles in different accessions, implying their contribution to drought stress resistance in cassava. Finally, the expression of twelve *MeNAC* genes was analyzed under osmotic, salt, cold, ABA, and H_2_O_2_ treatments, indicating that cassava *NACs* may represent convergence points of different signaling pathways. Taken together, this work found some excellent tissue-specific and abiotic stress-responsive candidate *MeNAC* genes, which would provide a solid foundation for functional investigation of the NAC family, crop improvement and improved understanding of signal transduction in plants. These data bring new insight on the complexity of the transcriptional control of *MeNAC* genes and support the hypothesis that NACs play an important role in plant growth, development, and adaption of environment.

## Introduction

The NAC family (NAM, no apical meristem; ATAF, Arabidopsis transcription activation factor; and CUC, cup-shaped cotyledon) is one of the largest groups of plant-specific transcription factors [1, 2, 3, 4]. NAM, the first *NAC* gene isolated from the petunia, plays an important role in determining positions of meristems and primordial [1]. ATAF1 and ATAF2, two *NAC* genes from Arabidopsis, have a negative effect on the plants’ response to biotic and abiotic stresses, respectively [5, 6, 7, 8, 9]. CUC2 (CUPSHAPED COTYLEDON 2), another *NAC* gene found in Arabidopsis, is considered to be vital for the development of embryos and flowers [2]. Typically, in the NAC protein family, there is a highly conserved N-terminal DNA-binding domain containing approximately 150 amino acids divided into five subdomains (A-E); nevertheless, the C-terminal region that contains the protein-binding activity domain is highly variable and plays an important role in the regulation of transcription [2, 10, 11, 12, 13].

There is abundant evidence indicating that NAC proteins play crucial roles in various aspects of plant growth and development, and adaption to the environment [14, 15], including maintenance of the shoot apical meristem [1, 16], cell division and expansion [17], nutrient remobilization [18], flower formation [19], lateral root development [20, 21], leaf senescence [22, 23, 24, 25, 26, 27], secondary cell wall biosynthesis [3, 28], fiber development [29], seed development [30], and response to pathogen infection [9, 12, 31, 32, 33, 34] and abiotic stresses [15, 26, 33, 35, 36, 37, 38, 39].

Additional studies have also confirmed that a large number of *NAC* genes induced by abiotic stresses play crucial roles in the regulation of plant tolerance to abiotic stress. Three Arabidopsis *NAC* genes (*ANAC019*, *ANAC055*, and *ANAC072*) showed up-regulation at transcription levels after drought, high salinity and abscisic acid (ABA) treatments, and those overexpression resulted in increased tolerance to drought [35]. Similarly, overexpression of *ATAF1* in the *Arabidopsis* enhanced tolerance to drought, ABA, salt, and oxidative stresses [14]. The effects of *NAC* genes on increasing tolerance to abiotic stress were also found in rice. *SNAC1*, an *NAC* gene in rice, can improve tolerance to drought and salt stresses in rice and transgenic plants were found to produce a 22–34% higher yield in the field under severe drought stress conditions [36]. Accordingly, *OsNAC10*-overexpressing rice plants showed an increased grain yield under both normal and drought conditions [37]. Overexpression of other three rice drought-responsive NAC genes (*OsNAC5*, *OsNAC6*, and *OsNAC10*) increased plant tolerance to drought and salt stresses [37, 40]. Therefore, NAC family genes are crucial regulators of plant tolerance to abiotic stress and crop yield.

To date, genome-wide analyses have identified a large number of NAC family members in several species with 152 *NAC* genes in *Nicotiana tabacum* [41], 117 *NAC* genes in the model plant *Arabidopsis thaliana* [13], 151 *NAC* genes in *Oryza sativa* [13], 163 *NAC* genes in *Populus trichocarpa* [42], 74 *NAC* genes in *Vitis vinifera* [43], 147 putative *NAC* genes in *Setaria italica* [44], 145 *NAC* genes in *Gossypium raimondii* [45], 167 *NAC* genes in *Musa acuminate* [46], and 71 *NAC* genes in *Cicer arietinum* [47]. However, no information is available for the *NAC* family in the cassava.

Cassava (*Manihot esculenta* Crantz) is the third most important crop, after rice and maize, in Africa, Asia, and Latin America and is considered a food security crop in these regions as the starchy roots provide nourishment for 800 million people around the world [48, 49]. Since this plant produces a high starch product at a minimum processing cost, cassava is also considered one of the major crops for bioethanol production [50, 51]. Cassava is particularly tolerant to drought and low-fertility soils under environmental stresses [49, 52]; however, the mechanisms underlying this tolerance to abiotic stress are less known. Therefore, an understanding of the molecular mechanisms underlying cassava tolerance to abiotic stress may provide effective ways for genetic improvement of stress tolerance of cassava and other crops. The high-quality sequencing data of cassava wild ancestors and cultivated varieties in our previous study provides an excellent opportunity for genome-wide analysis [53]. Based on the significance of NACs in the regulation of plant growth, development, and adaption to the environment, the NAC family was selected to perform a systematic analysis. In the present study, we identified 96 *NAC* genes from cassava and carried out detailed studies of their phylogeny, conserved motifs, gene structure, expression profiles in various tissues, and response to drought, osmotic, salt cold stresses and signaling of ABA and H_2_O_2_. Our results can provide a basis for future research on the evolutionary mechanisms and abiotic stress responses mediated by NACs in cassava.

## Materials and Methods

### Plant materials and treatments

W14 (*Manihot esculenta* ssp. *flabellifolia*) is an ancestor of the wild cassava subspecies which has a strong tolerance to drought stress. The South China 124 (SC124) is a widely planted cassava cultivar in China [52]. The Argentina 7 (Arg7) adapts to a geographical high-latitude region of Argentina [54]. All plants were grown in a glass house in the Chinese Academy of Tropical Agricultural Sciences (Haikou, China). The plants were grown from April to July 2013 during which time the temperature in the glass house ranged from 20 to 35°C. The transcripts of cassava *NAC* genes in different tissues were examined with wild subspecies (W14) and cultivated variety (Arg7) under normal growth conditions. Arg7, SC124, and W14 were chosen to study the transcriptional response of cassava NAC genes under drought stress. After two months of normal cultivation, the cassava seedlings similar in growth vigor were subjected to various treatments. For abiotic stress and signal molecule treatments, cassava seedlings were subjected to 200 mM mannitol for 24 d, 300 mM NaCl for 24 d, 3.27 M H_2_O_2_ for 72 h, 100 μM abscisic acid (ABA) for 72h, and low temperatures (4°C) for 48h following recovery, respectively.

### Identification and phylogenetic analyses of the NAC family in cassava

The whole protein sequence of cassava was obtained from the cassava genome database (http://www.phytozome.net/cassava.php). The Arabidopsis and rice NAC amino acid sequences were acquired from UniPort (http://www.uniprot.org/) and RGAP databases (http://rice.plantbiology.msu.edu/), respectively. To identify the cassava *NAC* family genes, the local Hidden Markov Model-based searches (HMMER: http://hmmer.wustl.edu/) were built from known NACs to search the cassava genome database [55]. Additionally, BLAST analyses with all the Arabidopsis and rice NACs as queries were employed to identify the predicted NACs in the cassava database. With the help of CDD (http://www.ncbi.nlm.nih.gov/cdd/) and PFAM databases (http://pfam.sanger.ac.uk/), all the potential cassava *NAC* genes identified from HMM and BLAST searches were accepted only if they contained the NAC domain; then multiple sequence alignments were used to confirm the conserved domains of predicted NAC sequences. Moreover, TMHMM Server ver.2.0 (http://www.cbs.dtu.dk/services/TMHMM/) was used to predict the membrane-bound MeNAC members. Finally, a bootstrap neighbor-joining (NJ) phylogenetic tree was constructed based on the multiple alignments of identified cassava NAC members with all the Arabidopsis NACs by Clustal X 2.0 and MEGA 5.0 [56, 57].

### Protein properties and sequence analyses

The molecular weight (MW) and isoelectric points (pI) of presumed NAC proteins were predicted by the online ExPASy proteomics server database (http://expasy.org/). The motifs were identified using MEME program (http://meme.nbcr.net/meme/cgi-bin/meme.cgi). The maximum number of motifs was 15 and the optimum width of motifs was set from 15 to 50 [58]. Furthermore, all identified motifs were annotated according to InterProScan (http://www.ebi.ac.uk/Tools/pfa/iprscan/). The information of each *MeNAC* gene in the genome was retrieved from the cassava database and the gene structures were identified using GSDS software (http://gsds.cbi.pku.edu.cn/). Exon/intron organization was further checked by alignment of coding sequence and genomic DNA sequence of each *NAC* gene.

### Transcriptomics analysis

The RNA-seq technique was employed to determine the expression of cassava *NAC* genes. Total RNA was extracted from stems, leaves, and roots in Arg7 and W14 under normal growth conditions and was also extracted from leaves and roots of Arg7, SC124, and W14 under normal conditions and 12 days drought treatment. Total RNA was isolated using the plant RNeasy extraction kit (TIANGEN, China) and the concentration and purity were evaluated using NanoDrop 2000c (Thermo Scientific, USA). Reverse transcription was implemented using 3 μg total RNA of each sample by RevertAid First Strand cDNA Synthesis Kit (Fermentas). According to the Illumina instructions, the cDNA libraries were constructed and subsequently subjected to sequencing by Illumina GAII following Illumina RNA-seq protocol. To obtain precise and reproducible results, each sample was replicated two times.

### Quantitative RT-PCR analysis

Expression of *MeNAC* genes in response to various abiotic stress (osmotic, salt, and cold) and related signaling (ABA and H_2_O_2_) were examined by qRT-PCR analysis with Stratagene Mx3000P Real-Time PCR system (Stratagene, CA, USA) using SYBR Premix Ex Taq (TaKaRa, Japan) according to the manufacturer’s instructions. The relative expression of the target genes was determined by the 2^–ΔΔCt^ method [59]. In order to obtain the optimal primer and template concentrations, a series of primer and template dilutions were performed prior to quantification experiments. Primers with high specificity and efficiency amplification on the basis of dissociation curve analysis and agarose gel electrophoresis are used to conduct quantification analysis (S8 Table). Moreover, to ensure the primer specificity, PCR products were sequenced. Amplification efficiencies of gene-specific primers ranged from 90% to 110%. EF1 and TUB that were verified to be constitutive expression were used as internal references for all the qRT-PCR analyses in this study [60]. Each treated sample contained a corresponding regularly-watered control and each sample had three independent biological replications. The treated and control plants at each time point were sampled for expression analysis. The relative expression levels of *MeNAC* genes in each treated time point were compared with that in each time point at normal conditions [61].

## Results and Discussion

### Identification and phylogenetic analysis of cassava NACs

Both BLAST searches and Hidden Markov Model searches were carried out to extensively identify cassava *NAC* genes from the cassava genome database using Arabidopsis and rice NAC sequences as queries. After these programs, 96 putative *NAC* gene family members, designated as *MeNAC1*-*MeNAC96*, were identified from the complete cassava genome. Conserved domain analysis further confirmed that all of the NACs contain the NAC domain or NAM domain at the N-terminus that are the basic characteristics of NAC family. When some cassava *NAC* genes contain alternative mRNA splicing, the complete sequence and its variant for each gene were used for further analyses. The 96 predicted NAC proteins ranged from 82 (MeNAC45) to 656 (MeNAC1) amino acid (aa) residues with an average of 342.7 aa, the relative molecular mass varied from 9.87 kDa (MeNAC45) to 74.79 kDa (MeNAC1), and the pIs ranged from 4.45 (MeNAC9 and MeNAC11) to 9.63 (MeNAC86) with 57 members showing pI <7 and others pI >7 (S1 Table). MeNAC9 shared a high degree (99%) of sequence identity with MeNAC11 based on amino acid sequence alignment. Additionally, MeNAC25 showed a deficiency of 55 or 56 amino acids at C-terminal relative to MeNAC9 and MeNAC11, suggesting that MeNAC25 might be a splice variant of MeNAC9 and MeNAC11. All of the cDNAs of identified *MeNAC* genes have been submitted to GenBank and their accession numbers in GenBank are shown in S2 Table.

**Fig 1:**
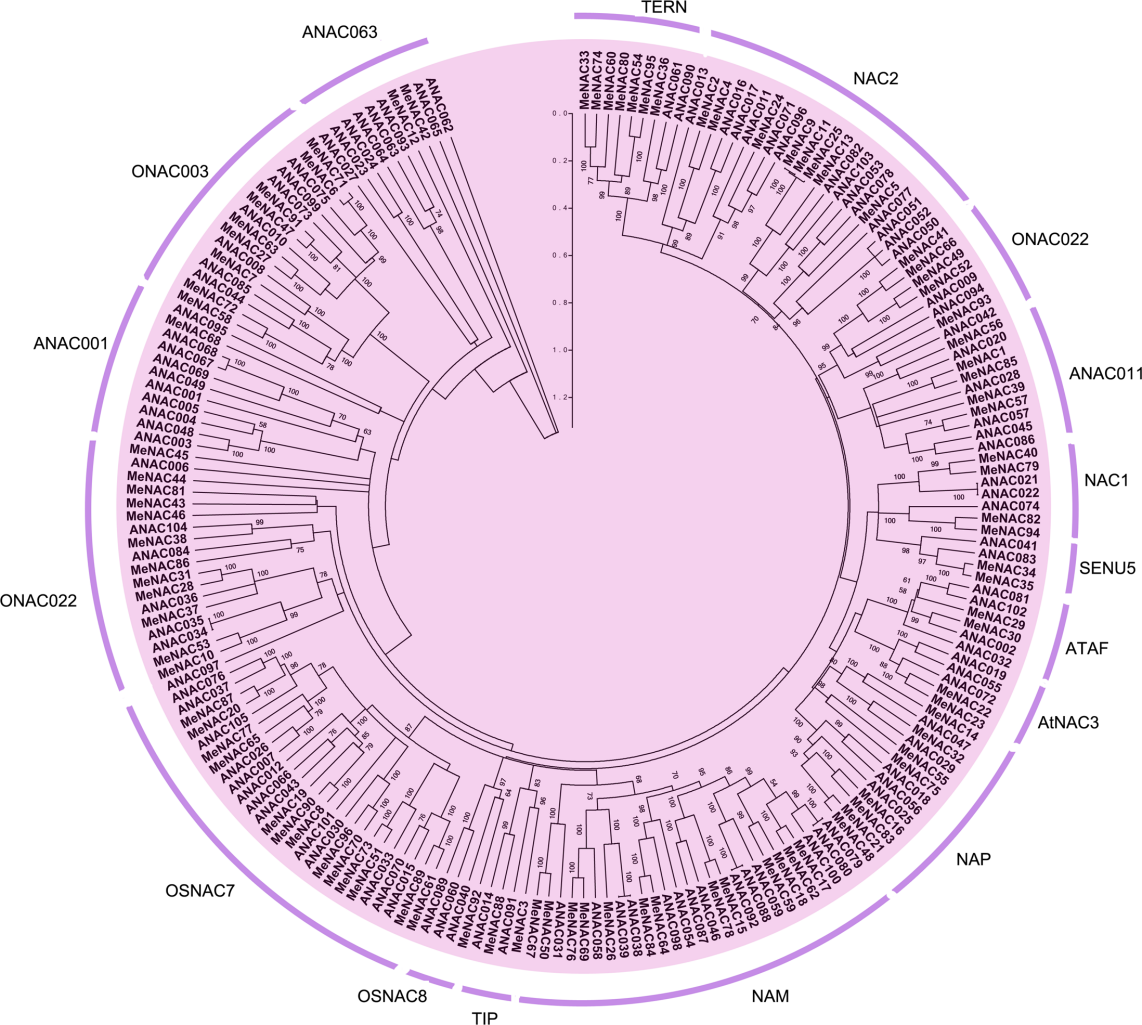
Phylogenetic analysis of NAC proteins from cassava and Arabidopsis. A total of 96 NACs from cassava and 105 NACs from Arabidopsis were used to construct the NJ tree with 1000 bootstrap based on the full length sequences of NACs. The NAC proteins are grouped into 16 distinct subgroups (TERN, NAC2, ONAC022, ANAC011, NAC1, SENU5, ATAF, AtNAC3, NAP, NAM, TIP, OSNAC8, OSNAC7, ANAC001, ONAC003 and ANAC063).“ANACs”are the NAC proteins from Arabidopsis. “MeNACs” indicate the NAC proteins from cassava.

To study the evolutionary relationships between cassava NAC proteins and known NACs from Arabidopsis, an unrooted Neighbor-Joining phylogenetic tree was created with the amino acids of NAC family proteins from cassava and Arabidopsis. The results indicated that 96 MeNACs can be divided into 16 subgroups together with their ANAC orthologs of Arabidopsis (Fig 1); however, phylogenetic analysis divided banana and soybean NACs into 8 and 6 subgroups, respectively [46, 62]. These data indicated that NAC proteins in cassava have greater diversity than that in these two species. Subgroup OSNAC8 and ANAC001 each only contain one MeNAC protein, while subgroup NAM and OSNAC7 each contain the maximum 13 MeNAC proteins. Similar to Arabidopsis and rice, these data identified the existence of a diversified MeNAC family in cassava with diverse functions [13, 63]. In addition, MeNAC9, MeNAC11 and their splice variant MeNAC25 exhibited close evolutionary relationship in subgroup NAC2.

Phylogenetic analysis also showed that there are some closely related orthologous NACs between cassava and Arabidopsis (MeNAC36 and ANAC061, MeNAC93 and ANAC042, MeNAC56 and ANAC020, MeNAC30 and ANAC002/ATAF1, MeNAC32 and ANAC029, MeNAC7 and ANAC008, and MeNAC68 and ANAC095), suggesting that an ancestral set of *NAC* genes existed prior to the divergence of cassava and Arabidopsis, and that NACs from cassava generally have a close relationship with the proteins from Arabidopsis. ANAC042, which showed a high degree of similarity with MeNAC93, has been reported to be involved in the regulation of camalexin biosynthesis, leaf senescence, oxidative, and heat stresses tolerance [26, 34, 38]. MeNAC30 shares high similarity with ANAC002/ATAF1 that has been shown to be involved in abiotic (drought, salt, and ABA) and biotic (necrotrophic pathogen *Botrytis cinerea*) stress responses, and the leaf senescence [14, 23]. MeNAC32 showed a high degree of similarity with ANAC029 that have recently been reported to play a role in tissue senescence [64]. These results suggest the possible functions of *NAC* genes in cassava.

### The membrane-associated MeNACs

It is well known that membrane-bound transcription factors (MTFs) of NAC family have vital role in biotic and abiotic stress response [65, 66, 67, 68]. Using TMHMM Server ver.2.0, 6 members (MeNAC1, -2, -3, -4, -88, and -92) were identified as membrane-associated MeNACs, designated as MeNTLs (NTM1-Like or ‘‘NAC with Transmembrane Motif 1”-Like) according to the name of membrane-bound NACs in Arabidopsis, of which MeNAC1 contains two TMHs, while other four MeNTLs contain one TMH (S3 Table). To date, comprehensive analyses have identified a large number of NAC MTFs in several species, including 18 NAC MTFs in *Arabidopsis thaliana* [66], 5 NAC MTFs in *Oryza sativa* [66], 11 NAC MTFs in *Glycine max* [62], 8 NAC MTFs in *Setaria italica* [44], 17 NAC MTFs in *Brassica rapa* [69], 8 NAC MTFs in *Cicer arietinum* [47], 8 NAC MTFs in *Zea mays* [70], and 7 NAC MTFs in *Brachypodium distachyon* [71]. According to the NJ phylogenetic tree (Fig 1), we found that MeNAC1, -2, -3, -4, -88 and -92 showed close phylogenetic relationship with ANAC028, -016, -091, -017, -014 and -040 respectively that were also identified as NAC MTFs in Arabidopsis [66]. This indicates that NAC MTFs in cassava might have similar function to their homologs in Arabidopsis. *ANAC016*, a senescence-associated NAC transcription factor, was reported to negatively regulate salt, drought and oxidative stress tolerance [25, 72]. However, *ANAC017* function on positively regulating drought stress tolerance [68]. These results suggest the possible functions of *MeNAC2* and *MeNAC*4 in response to abiotic stress.

### Gene structure and conserved motifs of cassava NACs

**Fig 2:**
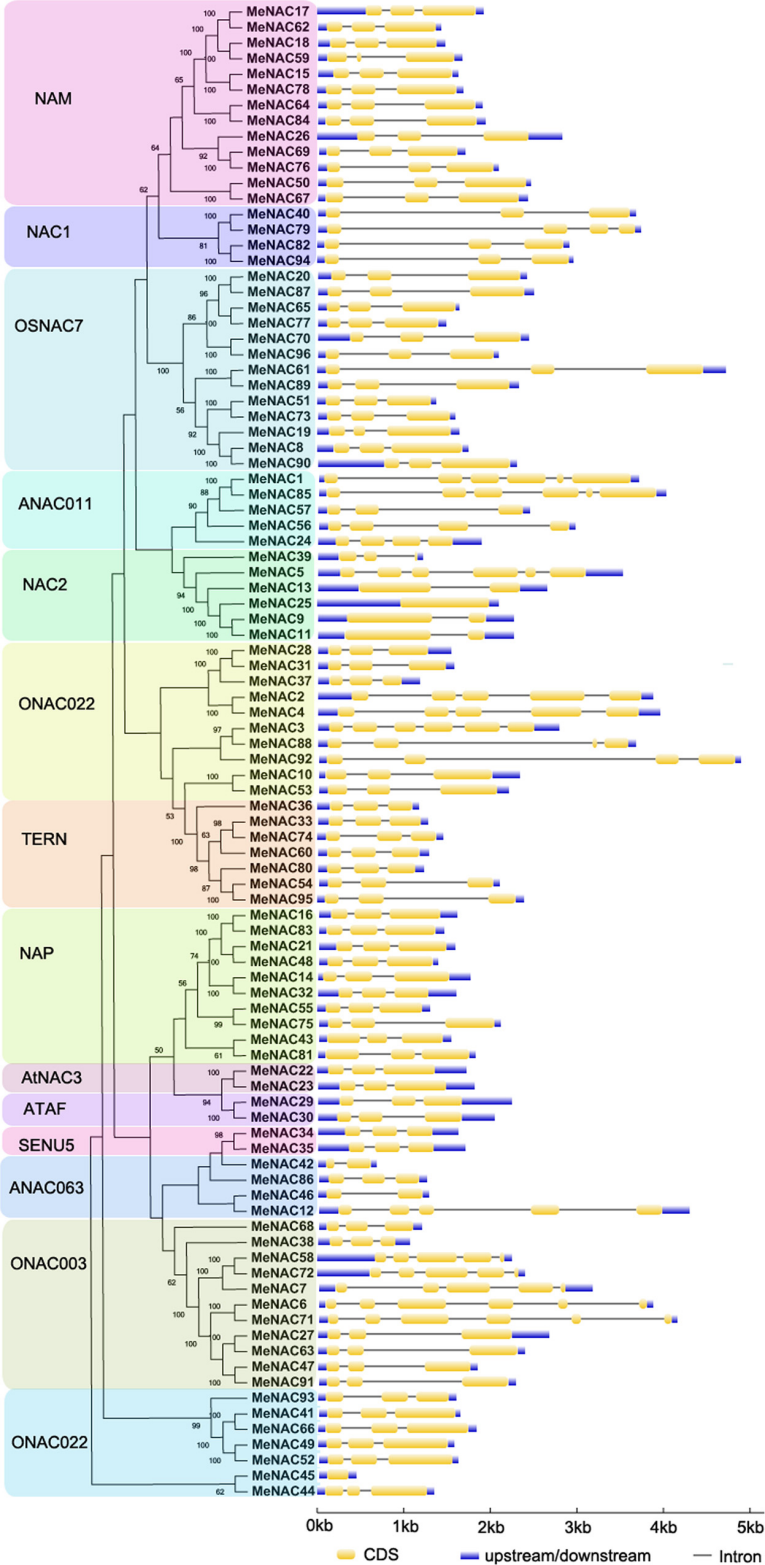
The exon-intron structure of *MeNAC* genes according to the phylogenetic relationship. The unrooted phylogenetic tree was constructed with 1000 bootstrap based on the full length sequences of MeNACs. Exon-intron structure analyses of *MeNAC* genes were performed by using the online tool GSDS. Lengths of exons and introns of each *MeNAC* gene were exhibited proportionally.

**Fig 3:**
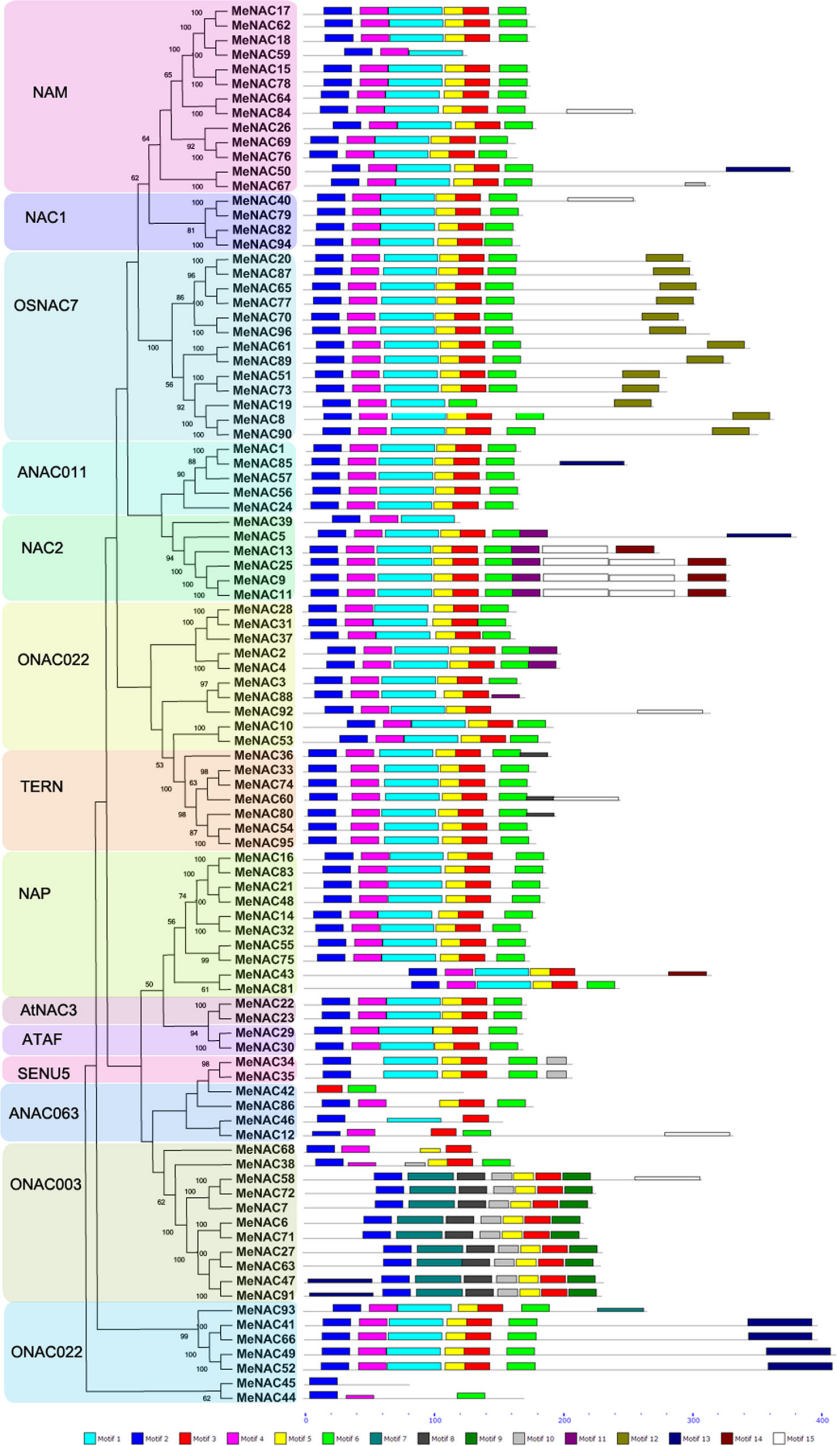
Conserved motifs of MeNAC proteins according to the phylogenetic relationship. The conserved motifs in the MeNAC proteins were identified by MEME. Grey lines represent the non-conserved sequences, and each motif is indicated by a colored box numbered at the bottom. The length of motifs in each protein was exhibited proportionally.

During the evolution of multigene families, gene structure was commonly diversified and thus could facilitate evolutionary co-option of genes for new functions to adapt to changes in the environment [45]. To further examine the structural features of cassava *NAC* genes, intron/exon distribution and conserved motifs were analyzed according to their phylogenetic relationships (Fig 2; Fig 3). Gene structure analysis indicated that the number of introns of *MeNAC* genes varied from 0 to 5 (Fig 2), which is similar to that in banana, where introns number vary from 0 to 6 [45]; however, the number of introns vary from 0 to 16 and 0 to 9 in rice and cotton, respectively [13, 46]. These results suggest a small gene structure diversity of cassava *NAC* genes compared with *NAC* genes in rice and cotton.

Additionally, we found that 72 of 96 *MeNAC* genes had two introns. This phenomenon was also observed in rice, cotton, and banana, with the majority of *NAC* genes containing two introns [13, 45, 46]. Two *MeNAC* genes (*MeNAC25* and *MeNAC45*) contained no intron in their open reading frame (ORF), while six *MeNAC*s (*MeNAC1*, *-85* from subgroup ANAC011, *MeNAC5* from subgroup NAC2, *MeNAC3* from subgroup ONAC022, and *MeNAC-6*, *-71* from subgroup ONAC003) had the maximum number of introns. According to Nuruzzaman et al. (2010) [13], the rate of intron loss is faster than the rate of intron gain after segmental duplication in rice. Thus, it is possible that the subgroups ANAC011, NAC2, ONAC022, and ONAC003 may represent the original genes. In addition, MeNAC9 and MeNAC11 contained one introns, whereas their splice variant MeNAC25 had no intron. This indicated that alternative splice led to the deficiency of C-terminal exon of MeNAC25. Generally, most of *MeNAC* members in the same group had similar exon-intron structure. This conserved intron numbers in each subfamily supports their close evolutionary relationship and the classification of subgroups.

To further examine the structural diversity of cassava NAC proteins, fifteen conserved motifs were predicted using the MEME program and subsequent annotation with InterPro (Fig 3; S1 Fig; S4 Table). All the MeNACs contain at least one of the four main motifs (motif 1, 2, 3, and 4) that were annotated as NAC domain and/or no apical meristem (NAM) domain; therefore, all the cassava NACs identified in this study have conserved features of the NAC family. Interestingly, most of the conserved motifs located in the N-terminal of NAC proteins are highly conserved for DNA-binding, indicating that these motifs may be essential for the function of NAC proteins; a similar phenomenon was also observed for currently identified NACs in potato [73]. Notably, the motifs in subgroup ANAC063 were the most diverse, which corresponds to the intron/exon distribution of this subgroup. Additionally, all of the cassava NAC proteins, except for MeNAC42, contained the motif 2. All of the MeNACs in subgroup OSNAC7 specially showed motif 12 and all of the subgroup ONAC003 MeNACs, except for the closely related genes MeNAC38 and MeNAC68, specifically show motif 7 and 9. Interestingly, MeNAC9, MeNAC11, and MeNAC25 showed similar constitution of conserved motifs, indicating their similar function. Generally, NAC proteins that were clustered in same subgroups shared similar motif composition, indicating functional similarities among members of the same subgroup.

### Expression profiles of *MeNAC* genes in different tissues

**Fig 4:**
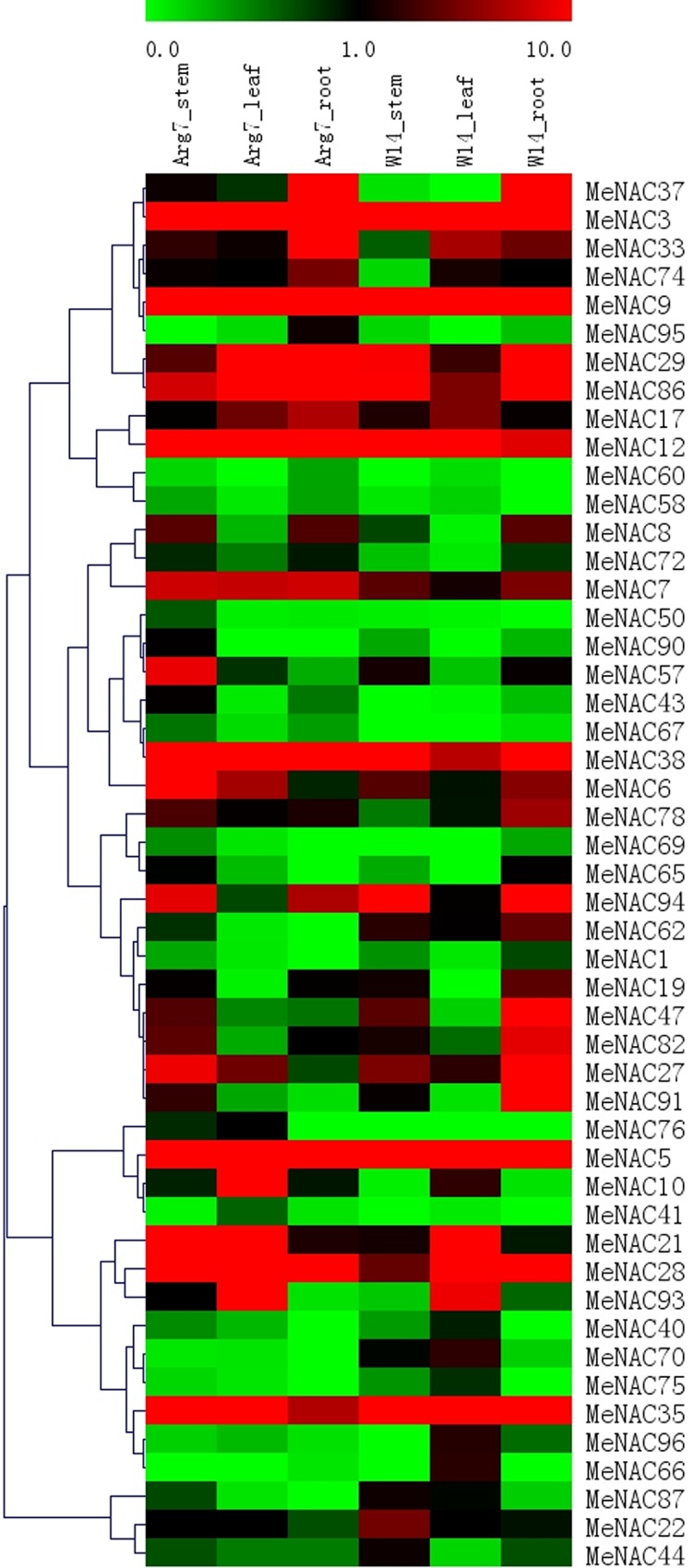
Expression profiles of *MeNAC* genes in different tissues of two cassava accessions. The transcript data generated from two replicates. The bar at the top of the heat map represents relative expression values.

To investigate the expression profiles of *NAC* genes in cassava development, the expression patterns of *MeNAC* genes in different tissues were analyzed using available transcriptome data. W14, a wild cassava subspecies, is the nearest ancestor of cultivated cassava with low tuber root yield, photosynthesis rate and starch content in root tubers, but strong tolerance to drought stress [53]. Arg7, a cultivated variety, contains excellent agronomic traits, such as maintaining growth under moderate drought stress [54]. Tissues of leaves, stems, and roots in W14 and Arg7 were sampled to explore the expression profiles of *MeNAC* genes, which will provide evidence for cassava development in wild subspecies and cultivated variety. Forty-nine of the 96 *MeNAC* genes had the corresponding transcripts data in the dataset, while the rest of the 47 *MeNACs* were not covered in the RNA-seq libraries (Fig 4; S5 Table). Of these 49 *MeNAC* genes, 29 (59%), 21 (43%), and 21 (43%) *MeNACs* had high expression levels (value >1) in stems, leaves, and roots of Arg7, respectively. The number of *MeNAC* genes with the high expression levels (value >1) in stems, leaves, and roots of W14 were 24 (49%), 23 (47%), and 26 (53%), respectively. Transcriptomic data also revealed that 28 *MeNAC* genes showed a constitutive expression pattern expressed in all the tissues of the two accessions, suggesting that these genes might play a role in plant growth and development [69]. On the contrary, the remaining 21 *MeNAC* genes exhibited differential expression patterns, specific to certain tissues, such as *MeNAC37*, *MeNAC10*, *MeNAC74*, and *MeNAC93*. This phenomenon was also observed in other plants, such as in Arabidopsis, rice, Chinese cabbage, and chickpea [12, 47, 69, 74]. Moreover, several reports have indicated that overexpression of tissue-specifically expressed *NAC* genes can promote the development of particular tissues, as *NAC1* from Arabidopsis promotes lateral root development [20], Arabidopsis *NARS1/NAC2* and *NARS2/NAM* are involved in embryogenesis [75], and rice *OsNAC5* and *OsNAC6* affects plant root growth [76, 77].

Some of the *MeNAC* genes showed higher expression levels at leaf and stem tissues in Arg 7 than that in W14. *MeNAC10*, *-29*, *-86*, *-38*, *-7* and *-6* had higher expression levels at leaf tissue in Arg7 than that in W14; *MeNAC78*, *-8*, *-57*, *-28*, *-21*, *-7*, *-6* and *-27* had higher expression levels at stem tissue in Arg7 than that in W14. However, some of the *MeNAC* genes had higher expression levels at roots in W14 than that in Arg7, including *MeNAC62*, *-78*, *-82*, *-94*, *-65*, *-19*, *-35*, *-6*, *-27*, *-47* and *-91*. The strong expression levels of these *MeNAC* genes in a special tissue in different accessions indicated their key roles in tissue development or function.

Overall, 11 out of 49 *MeNAC* genes showed high transcript abundance in all of the tested tissues of the two accessions, including *MeNAC17* in subgroup NAM, *MeNAC5*, *-9* in subgroup NAC2, *MeNAC28*, *-3* in subgroup ONAC022, *MeNAC29* in subgroup ATAF, *MeNAC35* in subgroup SENU5, *MeNAC86*, *-12* in subgroup ANAC063, and *MeNAC38*, *-7* in group ONAC003. The *NAC* genes with relative high transcripts in all the tested tissues could play a crucial role in the development of cassava. In contrast, *MeNAC69*, *-50*, *-67*, *-40*, -*65*, -*1*, -*60*, -*75*, -*58*, -*72*, -*41*, and *-66* had low expression levels in all of the tissues examined. Furthermore, we also observed that some closely related genes showed similar expression patterns; for example, *MeNAC50*, *-67* in subgroup NAM, *MeNAC58*, *-72* in subgroup ONAC003, and *MeNAC41*, *-66* in subgroup ONAC022 had weak expression in various tissues tested. Together, the tissue expression profiles of *NAC* genes in different accessions provide important evidence for further investigation of cassava development.

### Expression analysis of *MeNAC* genes in response to drought in different accessions

**Fig 5:**
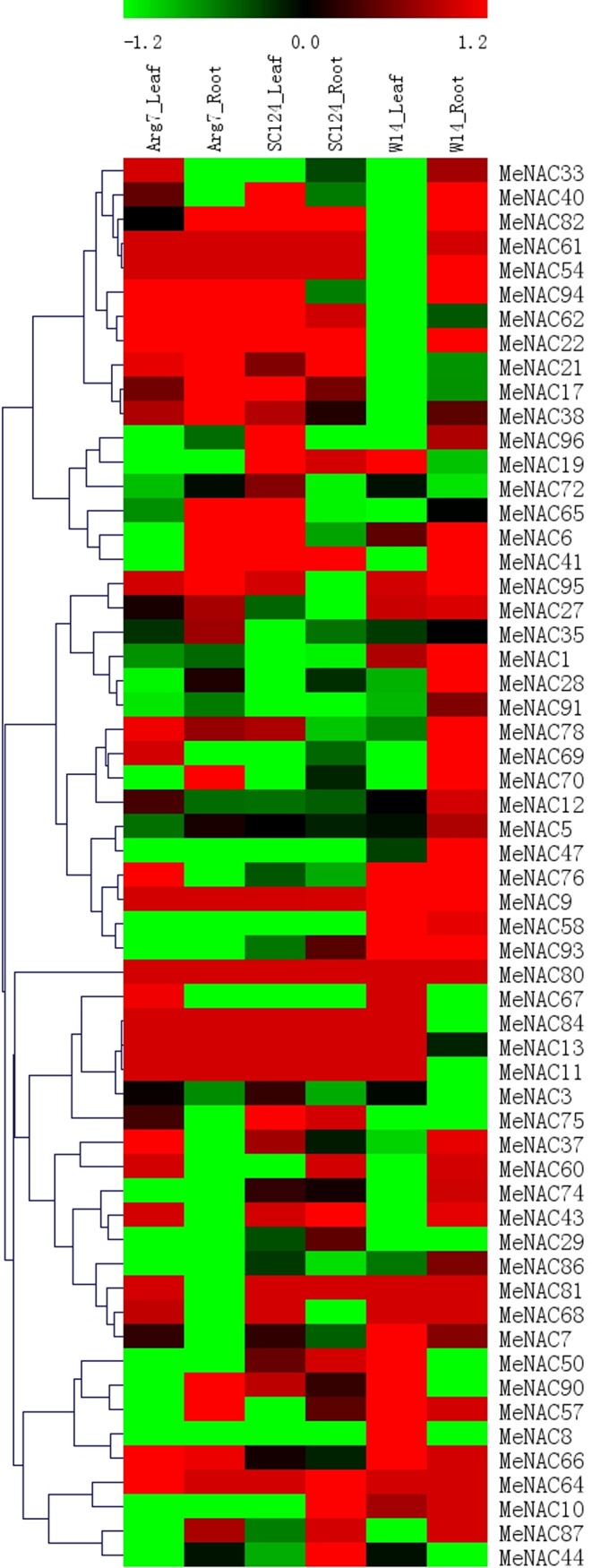
Expression profiles of *MeNAC* genes in leaves and roots of three cassava accessions after drought treatment. The transcript data generated from two replicates. The relative expression values were log_2_ transformed. The bar at the top of the heat map represents relative expression values.

Previous studies revealed that *NAC* family genes play an important role in plants’ response to drought or osmotic stress [14, 35, 36, 37, 40, 68, 78]. Genome-wide expression analysis of *NAC* genes in response to drought can provide an opportunity to further understand the mechanisms involved in cassava’s strong tolerance. In order to identify the expression profiles of cassava *NAC* genes in response to drought stress, three-month-old cassava seedlings (a wild subspecies W14 and two cultivated varieties Arg7 and SC124) were deprived of water for 12 days and then the leaves and roots tissues were collected to extract RNA for subsequent RNA-seq analysis. Fifty-eight of the 96 *MeNACs* had the corresponding transcripts data within the dataset (Fig 5; S6 Table). In the Arg7 variety, 32% and 36% of *MeNAC* genes showed induction by drought stress in leaves and roots, respectively. In the SC124 variety, 39% and 29% of *MeNAC* genes were transcriptionally induced after drought treatment in leaves and roots, respectively. In the W14 subspecies, 26% and 52% of *MeNAC* genes were up-regulated by drought stress in leaves and roots, respectively. These results suggest that the total number of *NAC* genes induced by drought was more in W14 than that in Arg7 and SC124, indicating the comprehensively transcriptional response of *NAC* genes responding to drought in W14 subspecies. W14 exhibited stronger tolerance to drought than SC124 and Arg7, two varieties commonly cultivated in China and Southeast Asia, respectively [53]. Moreover, numerous studies have confirmed that the *NAC* family genes play a positive role in the drought stress response in various species [14, 35, 36, 37, 40, 68, 78, 79, 80, 81]; therefore, the high ratio of *NAC* members transcriptionally induced by drought in W14 subspecies might contribute to its strong tolerance to drought. Additionally, from the previously describe transcriptomic data, we also observed that the number of *NAC* genes up-regulated by drought at transcription levels was significantly greater in roots than that in leaves in W14. Cassava can form deep root systems (below 2 m soil depth), which is beneficial for penetrating into deeper soil layers and absorbing water stored in the soil [82]. Thus, cassava *NAC* genes might play a regulatory role in water uptake from soil by roots, hence maintaining strong tolerance to drought stress in the W14 subspecies.

Generally, *MeNAC* genes showed similar expression profiles for leaves or root tissues in Arg7 and SC124, which differs from W14. Transcripts of some *MeNAC* genes (*MeNAC69*, *MeNAC76*, *MeNAC40*, *MeNAC1*, *MeNAC37*, *MeNAC33*, *MeNAC58*, *MeNAC7*, *MeNAC47*, and *MeNAC91)* were up-regulated in the roots of W14, but down-regulated in the roots of SC124 and Arg7 after drought treatment. The expression of some *MeNAC* genes, such as *MeNAC8*, *MeNAC1*, *MeNAC57*, *MeNAC10*, *MeNAC58*, and *MeNAC93*, increased in leaves of W14, but decreased in leaves of SC124 and Arg7 after drought treatment. The differential response of *NAC* genes to drought in different accessions implied that mechanisms involved in the *NAC-*mediated drought response are different between wild subspecies and cultivated varieties. Notably, although some *MeNAC* genes showed close phylogenetic relationships, they exhibited different responses to drought at transcriptional levels, such as *MeNAC69*, *-76* in subgroup NAM, *MeNAC82*, *-94* in subgroup NAC1, *MeNAC96*, *-70* in subgroup OSNAC7, and *MeNAC11*, *-9* in subgroup NAC2. Taken together, the transcriptional response of *MeNAC* genes to drought stress in wild subspecies and cultivated varieties will lay a foundation for further investigation of the underlying mechanisms of strong drought tolerance in cassava.

### Expression profiles of *MeNAC* genes with the treatments of various stress and related signaling

Accumulating evidence indicates that *NAC* genes play an important role in the regulation of plant tolerance to various stressors and related signaling transduction in various species [47, 70, 83, 84]. Thus, 12 *MeNAC* genes (*MeNAC9*, -*12*, *-22*, *-29*, *-35*, *-38*, *-57*, *-61*, *-64*, *-66*, *-80*, and *-94*) distributed in different subfamilies and up-regulated in some tissues or in a special tissue by drought stress, according to our RNA-seq data in different cassava accessions, were selected to further examine their response to osmotic, salt, cold, ABA, and H_2_O_2_ treatments.

**Fig 6:**
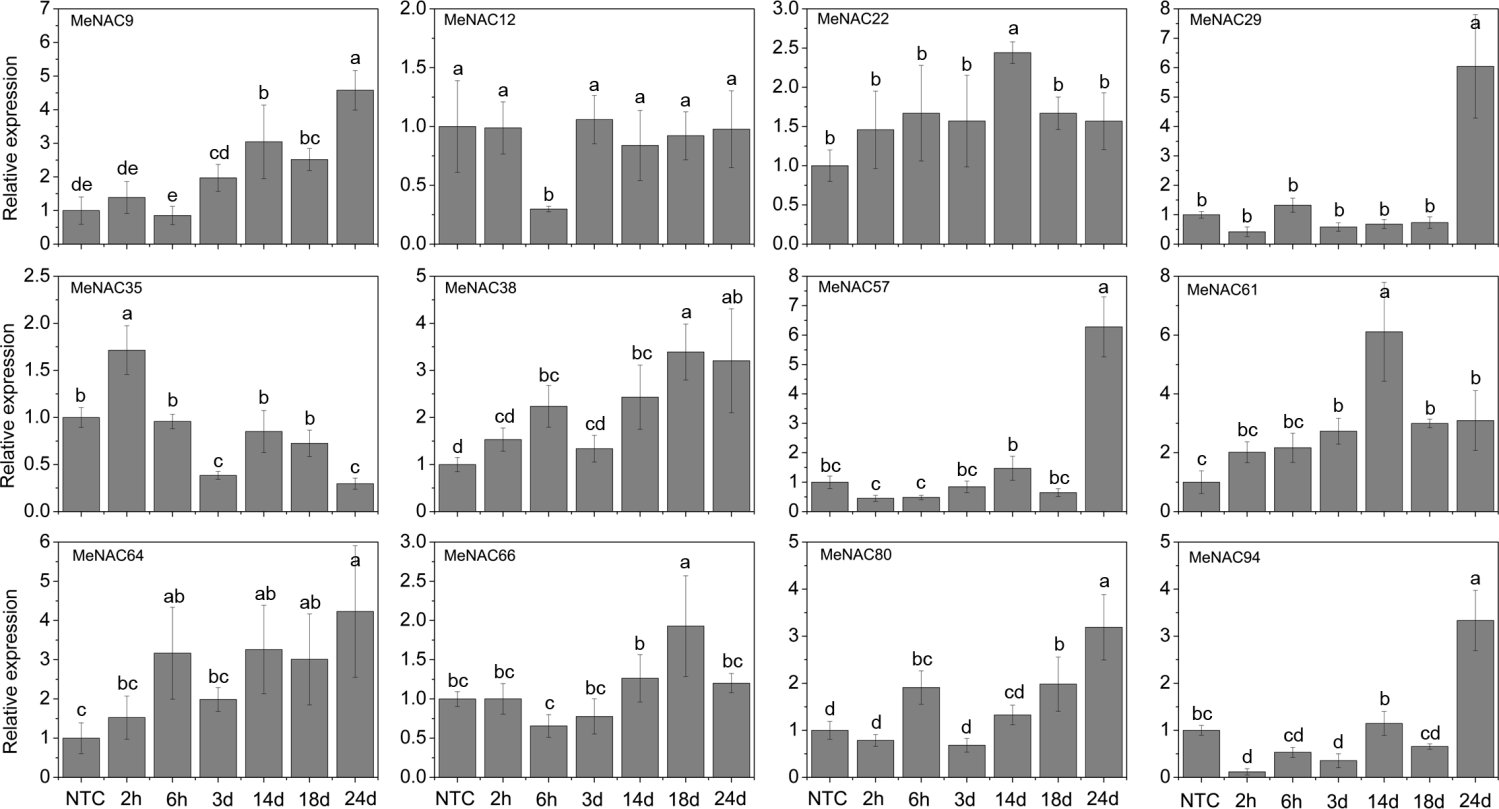
Expression profiles of *MeNAC* genes in leaves under osmotic stress. The relative expression levels of *MeNAC* genes in each treated time point were compared with that in each time point at normal conditions. NTC (no treatment control) at each time point was normalized as “1”. Data are means ± SE calculated from three biological replicates. Means denoted by the same letter do not significantly differ at *P* <0.05 as determined by Duncan’s multiple range test.

Under osmotic treatment, *MeNAC22*, *-38*, *-61* and -*64* showed up-regulation at all treated time-points, among which *MeNAC38*, *-61* and -*64* showed significant induction at 14d, 18d and 24d. *MeNAC9*, *-66* and *-80* expression was induced during 14–24d treatment, in which *MeNAC9* and *-80* showed significant induction at 18d and 24d. *MeNAC29*, *-57* and *-94* were significantly induced at 24d treatment. *MeNAC12* and *-35* showed obviously down-regulation at 6h, 3d or 24d treatments (Fig 6). Notably, *MeNAC61* showed up-regulation at all treated time-points and reached the highest expression level (value >6) at 14d, indicating their possible roles in osmotic/drought stress responses. *ANAC002/ATAF1*, *ANAC017*, *ANAC019*, *ANAC055*, *ANAC072*, and *ANAC096* have been reported to positively regulate drought stress tolerance in Arabidopsis [14, 35, 68, 78]. *MeNAC22*, an orthologue of *ANAC072* that is strongly induced by osmotic and drought stress, may represent a functional gene conferring tolerance to drought in cassava. However, some *NAC* genes, including *ANAC016* and *ANAC053/NTL4*, which showed significant up-regulation during dehydration, were reported to negatively regulate drought stress tolerance [72, 85]. Thus, we concluded that the roles of these negative regulators are also important to fine-tune drought stress responsive pathway together with positive regulators. In rice, four *NAC* genes (*SNAC1*, *SNAC2/OsNAC6*, *OsNAC5*, and *OsNAC10*) have been reported to show induction under drought treatment and function as positive factors in the regulation of plant tolerance to drought/osmotic stress [33, 36, 37, 40, 86]. In maize, 8 *NAC* genes (*ZmNAC18*, *-51*, *-52*, *-72*, *-73*, *-75*, *-99*, and *-145*) were found to be up-regulated by desiccation treatment in the tolerant genotype [70]. In chickpea, 14 out of 23 *CaNACs* (*CaNAC05*, *-06*, *-16*, *-19*, *-21*, *-27*, *-31*, *-40*, *-41*, *-43*, *-47*, *-50*, *-57*, and *-67*) were up-regulated under dehydration [47]. Together, these results indicate the important roles of these *NAC* genes in response to osmotic/drought stress.

**Fig 7:**
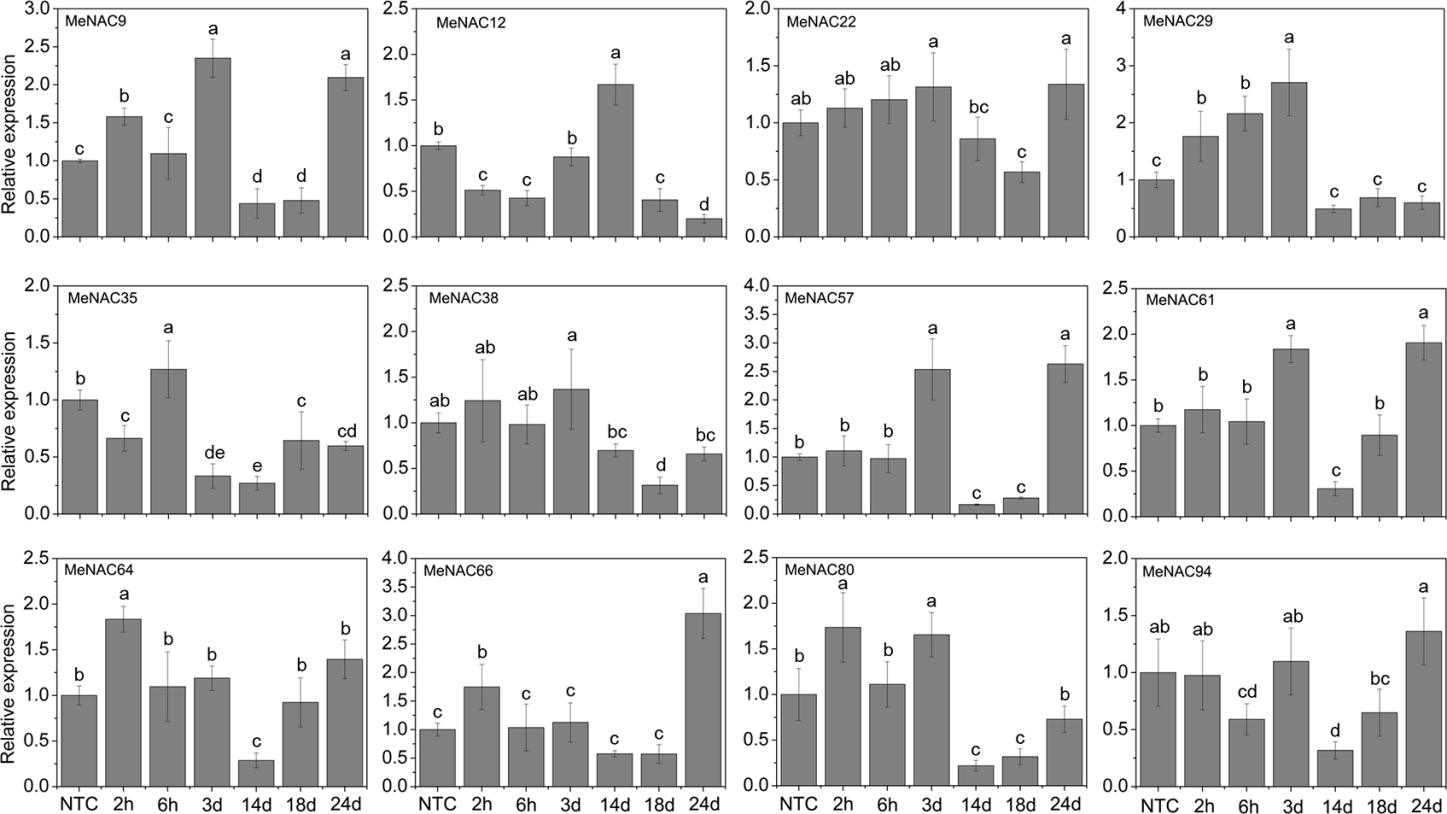
Expression profiles of *MeNAC* genes in leaves under salt stress. The relative expression levels of *MeNAC* genes in each treated time point were compared with that in each time point at normal conditions. NTC (no treatment control) at each time point was normalized as “1”. Data are means ± SE calculated from three biological replicates. Means denoted by the same letter do not significantly differ at *P* <0.05 as determined by Duncan’s multiple range test.

As shown in Fig 7, under NaCl treatment, *MeNAC9*, -*22*, *-61*, *-64*, and *-66* were induced after 2h-3d and 24d treatment with *MeNAC9* and *-61* showing significant up-regulation at 3d and 24d and *MeNAC64* and *-66* at 2h and 24d. *MeNAC29* and *MeNAC80* were induced after 2h-3d treatment. *MeNAC12* and *-35* showed significant up-regulation at 14d and 6h, respectively. *MeNAC38*, *-57*, and -*94* were obviously down-regulated at 18d, 14d-18d and 14d, respectively. In Arabidopsis, some NAC genes, including *ANAC002/ATAF1*, *ANAC062*, *ANAC016*, *ANAC019*, *ANAC055*, *ANAC069/NTM2*, *ANAC072/RD26*, *ANAC083/VNI2*, *ANAC092/AtNAC2*, and *NTL8/ANAC040* were reported to be up-regulated at transcriptional levels after salt treatment [14, 21, 24, 25, 65, 87, 88, 89, 90]; accordingly, 21 rice *NAC* genes (*SNAC1*, *ONAC09*/*OsNAC5*, *-06*, -*10*, *-11*, *-15*, *-27*, *-28*, *-39*, *-45*, *-59*, *-60*, *-67*, *-68*, *-73*, *-74*, *-85*, *-88*, *-103*, *-122*, *-132*, and *-139*) showed up-regulation with the treatment of salt stress [36, 63, 91]. Conversely, 39 rice *NAC* genes were down-regulated after salt treatment [13]. Evidence has suggested a positive role of some *NAC* genes in response to salt stress, such as *ANAC083*, *SNAC1*, *ONAC09/OsNAC5*, *OsNAC10*, and *ONAC045* [36, 91, 92, 37, 24]. On the other hand, other NACs, including *ANAC016*, *ANAC062*, *ANAC069*, and *AtNAC2/ANAC092* act as negative regulators in salt stress response in Arabidopsis [25, 67, 90, 93]. These studies indicated that NAC family genes may be positively or negatively involved in the salt stress response.

**Fig 8:**
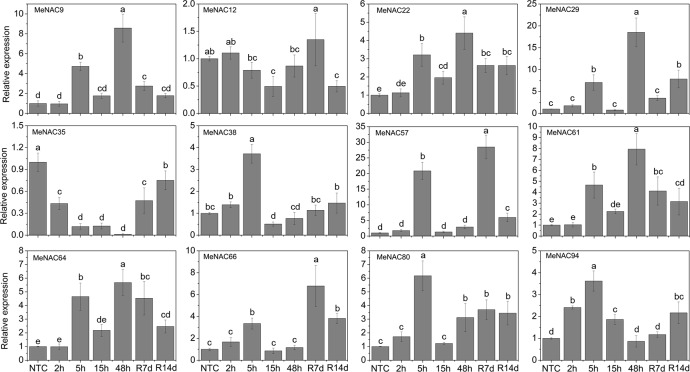
Expression profiles of *MeNAC* genes in leaves under cold stress. The relative expression levels of *MeNAC* genes in each treated time point were compared with that in each time point at normal conditions. NTC (no treatment control) at each time point was normalized as “1”. Data are means ± SE calculated from three biological replicates. Means denoted by the same letter do not significantly differ at *P* <0.05 as determined by Duncan’s multiple range test.

Cold stress is a major environmental factor that affects plant growth and crop productivity [94]. The mechanisms involved in NACs conferring cold tolerance are largely unknown. In response to cold stress, numerous physiological and molecular changes occur, such as an increase in the levels of metabolites and induction of the expression of some cold responsive genes [94]. In Arabidopsis, *NTL4*/*ANAC053 NTL7*/*ANAC017* were induced by cold stress [65]. In rice, 16 *NAC* genes (*ONAC007*, *-10*, *-15*, *-27*, *-28*, *-39*, *-45*, *-59*, *-67*, *-68*, *-73*, *-74*, *-85*, *-103*, *-122*, and *-132*) showed up-regulation under cold treatment [63]. In Chinese cabbage, 5 *BrNAC* genes (*Bra000192*, *Bra001000*, *Bra011037*, *Bra003244*, and *Bra026595*) were up-regulated under cold treatment [69]. Under cold treatment following recovery, *MeNAC9*, *-22*, -*57*, *-61*, -*64*, and *-80* showed up-regulation at all the treated time-points, among which *MeNAC9*, *-22*, -*61*, and *-64* showed significant up-regulation at 5h and 48h and *MeNAC57* and *-80* was significantly induced at 5h, R7d, and R14d. *MeNAC29* and *-66* were significantly up-regulated at 5h and R14d. *MeNAC38* and *MeNAC94* were up-regulated during 2–5h cold treatment and following recovery. *MeNAC35* expression was repressed during all the treated time points (Fig 8). *MeNAC57*, the most highly induced genes (over 20-fold at two time-points), could be used in further functional characterization. Cassava, an important tropical crop, distributes in tropical areas of the world. Low temperatures limit agricultural productivity and the development of cassava. Research on the *NAC*-mediated cold response in cassava will benefit further functional characterization of *NAC* genes and investigations of the mechanisms underlying the cold response in cassava.

**Fig 9:**
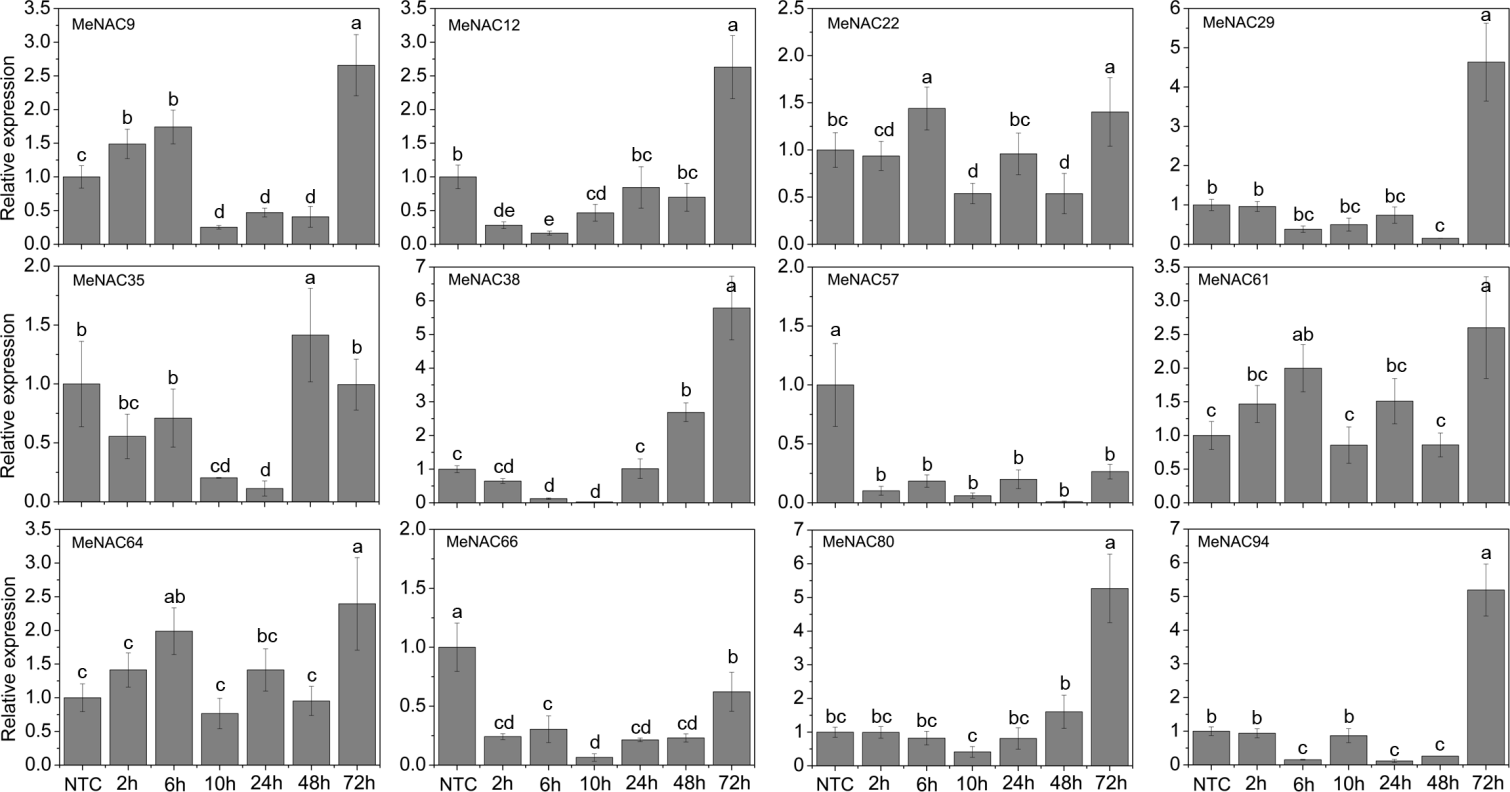
Expression profiles of *MeNAC* genes in leaves under ABA treatment. The relative expression levels of *MeNAC* genes in each treated time point were compared with that in each time point at normal conditions. NTC (no treatment control) at each time point was normalized as “1”. Data are means ± SE calculated from three biological replicates. Means denoted by the same letter do not significantly differ at *P* <0.05 as determined by Duncan’s multiple range test.

The phytohormone ABA plays a crucial role in plant development, regulation of stomatal behavior and responses to abiotic stress, such as salinity, drought, and cold [95]. Accumulated evidence has shown that NACs are involved in ABA-mediated signal transduction in plants [14, 36, 37]. In Arabidopsis, several NACs, including *ANAC002/ATAF1*, *-016*, *-053*, *-083/VNI2*, *-019*, *-029*, -0*55*, *-072*, *-096*, -*010*, *-012*, *-040*, *-062* and *-009* have been shown to regulate ABA-mediated processes [21, 24, 64, 67, 72, 78, 85, 89]. To investigate the involvement of NAC genes in ABA signaling, we examined the expression of 12 *NAC* genes in response to ABA treatment. The results showed that *MeNAC9*, *-61*, and *-64* expression were induced during 2h-6h and 72h treatments with significant up-regulation at 72h and *MeNAC12*, *-29*, *-38*, *-80*, and *-94* was also significantly induced at 72h treatment, whereas *MeNAC57* and *MeNAC66* were strongly repressed at all the treated time-points and *MeNAC35* was obviously down-regulated during 10h-24h treatments. The expression of *MeNAC22* showed no obvious trend after ABA treatment (Fig 9). The response of cassava *NAC* genes to ABA treatment suggested the possible roles of *MeNAC* genes in ABA signaling.

**Fig 10:**
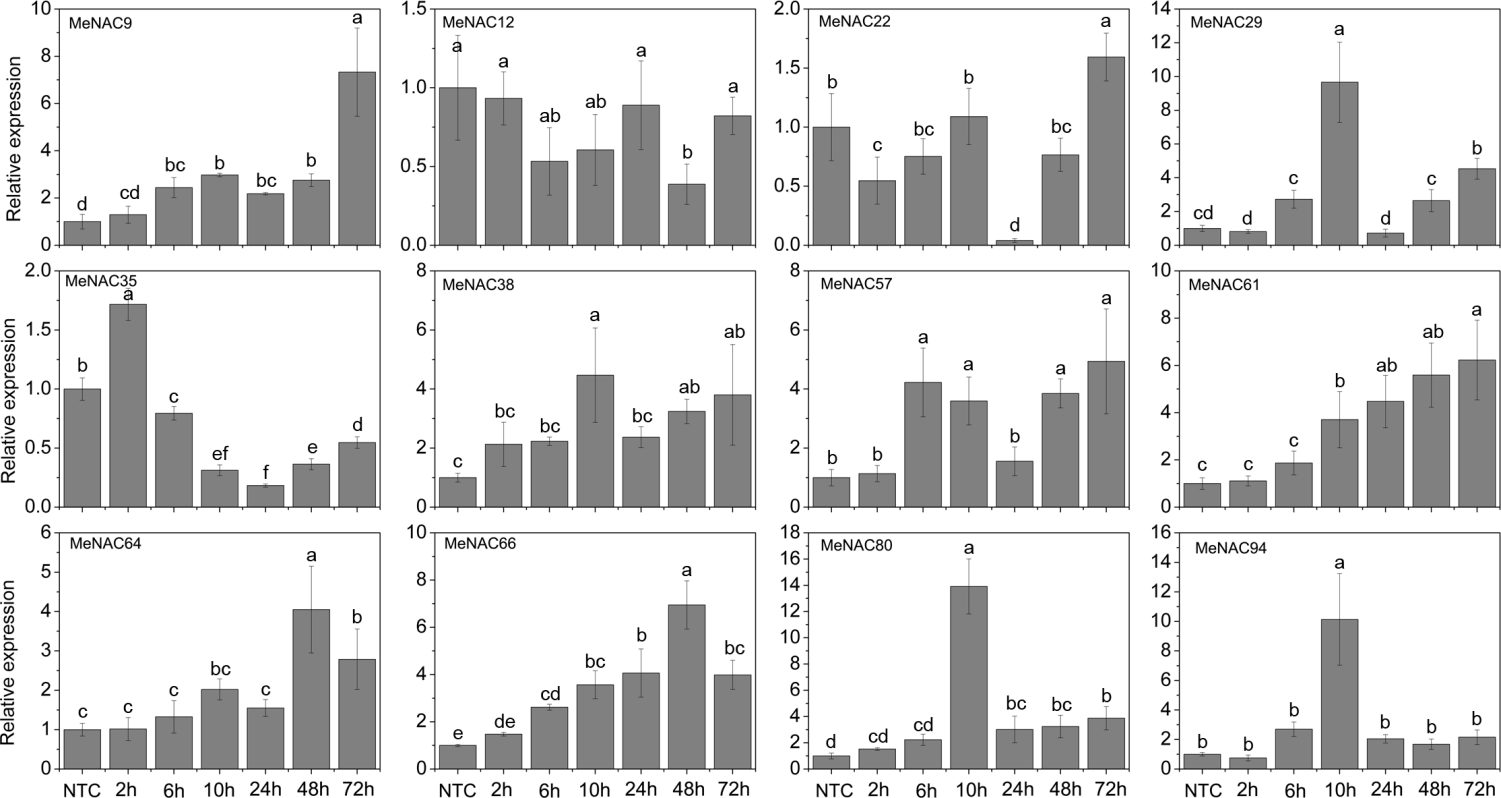
Expression profiles of *MeNAC* genes in leaves under H_2_O_2_ treatment. The relative expression levels of *MeNAC* genes in each treated time point were compared with that in each time point at normal conditions. NTC (no treatment control) at each time point was normalized as “1”. Data are means ± SE calculated from three biological replicates. Means denoted by the same letter do not significantly differ at *P* <0.05 as determined by Duncan’s multiple range test.

H_2_O_2_ is considered a specific component of several biotic and abiotic signaling pathways and its accumulation has been found to be induced by environmental and developmental stimuli [96]. In Arabidopsis, some evidence has suggested that *NAC* genes play a positive role in response to oxidative stress; for example, overexpression of *ANAC013* in *Arabidopsis* increased tolerance to oxidative stress, with more fresh weight under methyl viologen and rotenone treatments [97]. *ANAC042* can modulate cellular H_2_O_2_ levels, thus improving the ROS balance and extending longevity and increasing tolerance to heat stress [26, 38]. *ANAC059/ORS1* was found to be rapidly induced by H_2_O_2_ treatment and function on controlling senescence in Arabidopsis [98]. On the other hand, other NAC TFs, such as *ANAC016*, act as negative regulator in oxidative stress response [25].To determine whether cassava *NAC* genes play a role in H_2_O_2_ signaling pathways, we analyzed the expression of 12 *MeNAC* genes in response to H_2_O_2_. The results suggested that *MeNAC9*, -*38*, -*57*, -*61*, -*64*, *-66*, and *-80* showed induction during 2h-72h treatments with significant induction at 48h and 72h. In addition, *MeNAC29* and *MeNAC94* were significantly up-regulated during 6h-10h H_2_O_2_ treatment. H_2_O_2_ treatment also caused a seriously decrease in transcription levels of *MeNAC22* and *MeNAC35* at 24h (Fig 10). The expression levels of *MeNAC9*, -38, -61, and -66 increased with prolonging of treatment time, suggesting their possible function in H_2_O_2_ signaling. These results suggest that cassava *NACs* are likely involved in the H_2_O_2_ signaling pathway.

**Fig 11:**
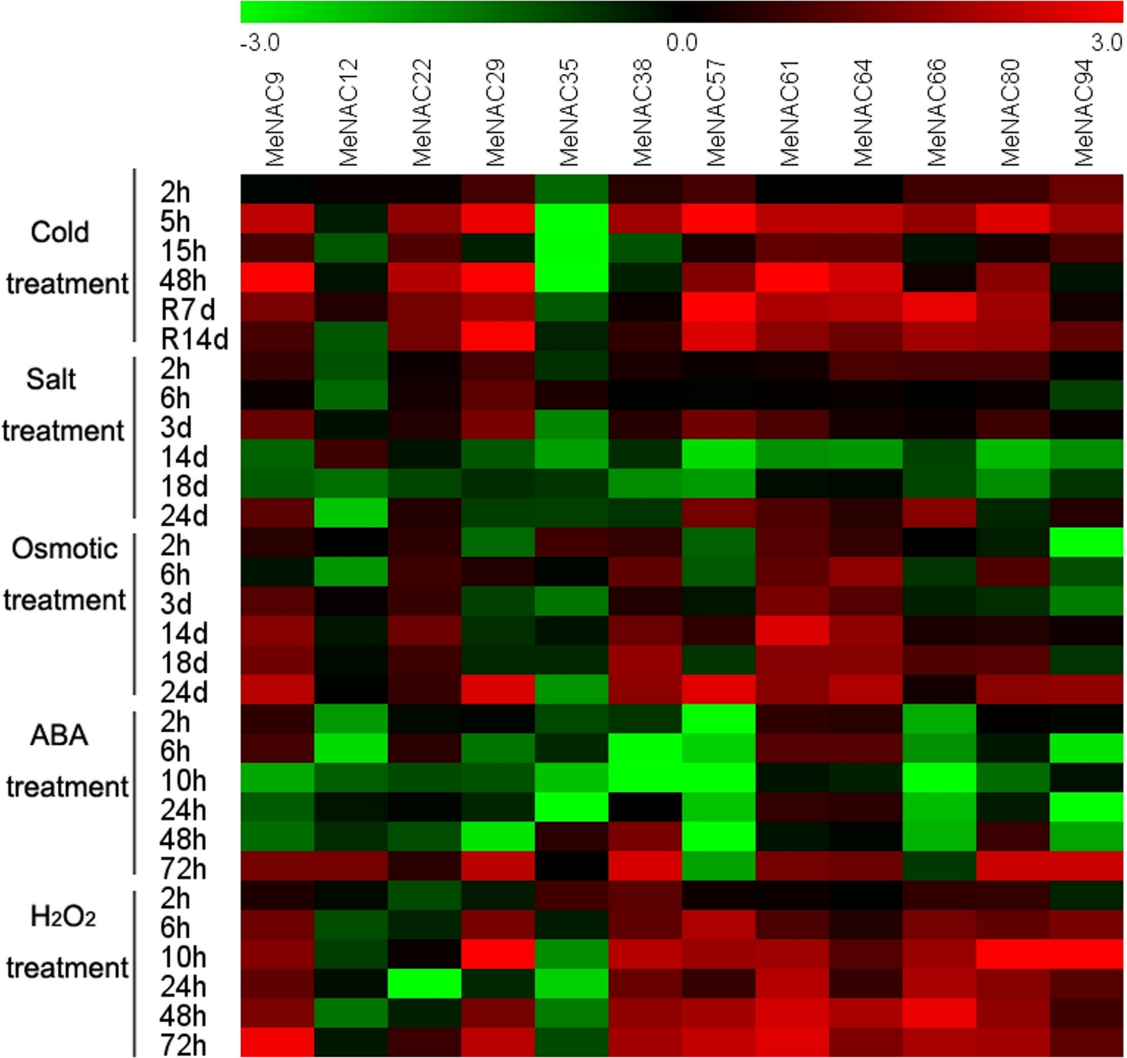
Expression profiles of *MeNAC* genes in leaves under various stresses, ABA and H_2_O_2_ treatments. Log2 based values from three replicates of qRT-PCR data were used to create the heatmap. The scale represents the relative signal intensity values. Relative expression values for each gene after various treatments are provided in Figs 6–10 and S7 Table.

Taken together, the expression profiles of *MeNAC* genes under various conditions suggest that different *MeNAC* genes may participate in different signaling or stress responses, and that a single *MeNAC* gene is also involved in multiple signaling or stress processes. Moreover, most of the cassava *NAC* genes could be significantly induced by multiple stressors, ABA, and H_2_O_2_ treatments, indicating that *NAC* genes are important components of multiple transduction pathways in cassava (Fig 11; S7 Table).

In conclusion, we identified 96 *NAC* genes from the cassava genome and established their basic classification and evolutionary characteristics, which will provide gene resources for functional characterization of *NAC* genes. The differential expression patterns of *MeNACs* in tissues of the wild subspecies and cultivated varieties suggested that they play different roles in cassava development, thus assisting in understanding the molecular basis for genetic improvement of cassava. Additionally, transcriptomic analysis of different cassava accessions associated with drought stress revealed that a high rate of *NAC* members in the W14 subspecies were induced by drought, which may contribute to its strong tolerance to drought. Furthermore, expression analysis of *MeNAC* genes after various treatments suggested that they have a comprehensive response to osmotic, salt, cold, ABA, and H_2_O_2_, implying that cassava NACs may represent convergence points of different signaling pathways. These data will benefit further investigation of NACs mediated signaling transduction pathways. Overall, this work provides a solid foundation for functional investigation of the NAC family, crop improvement, and an improved understanding of signal transduction in plants.

## Supporting Information

S1 Table Characteristics of MeNAC family in cassava. (XLS)

S2 Table The accession numbers of NACs in cassava and Arabidopsis. (XLS)

S3 Table Putative membrane-bound cassava NACs. (XLSX)

S4 Table Conserved amino acid motifs and annotation of MeNACs. (XLS)

S5 Table The expression data of the cassava *NAC* genes in different tissues. (XLSX)

S6 Table The expression data (log2-based values) of the cassava *NAC* genes after drought treatment. (XLS)

S7 Table The expression data (log2-based values) of the cassava *NAC* genes after various stresses, ABA and H_2_O_2_ treatments. (XLS)

S8 Table Primers used in qRT-PCR analysis. (XLS)

S1 Fig Sequence logos for conserved motifs identified in MeNACs by MEME analysis. (TIF)
