# Supplementary figures and images for: Genome-Wide Identification and Expression Analysis of the NAC Transcription Factor Family in Cassava
Source: PLoS One. 2015 Aug 28;10(8):e0136993. doi: 10.1371/journal.pone.0136993 (PMC4552662; doi:10.1371/journal.pone.0136993)

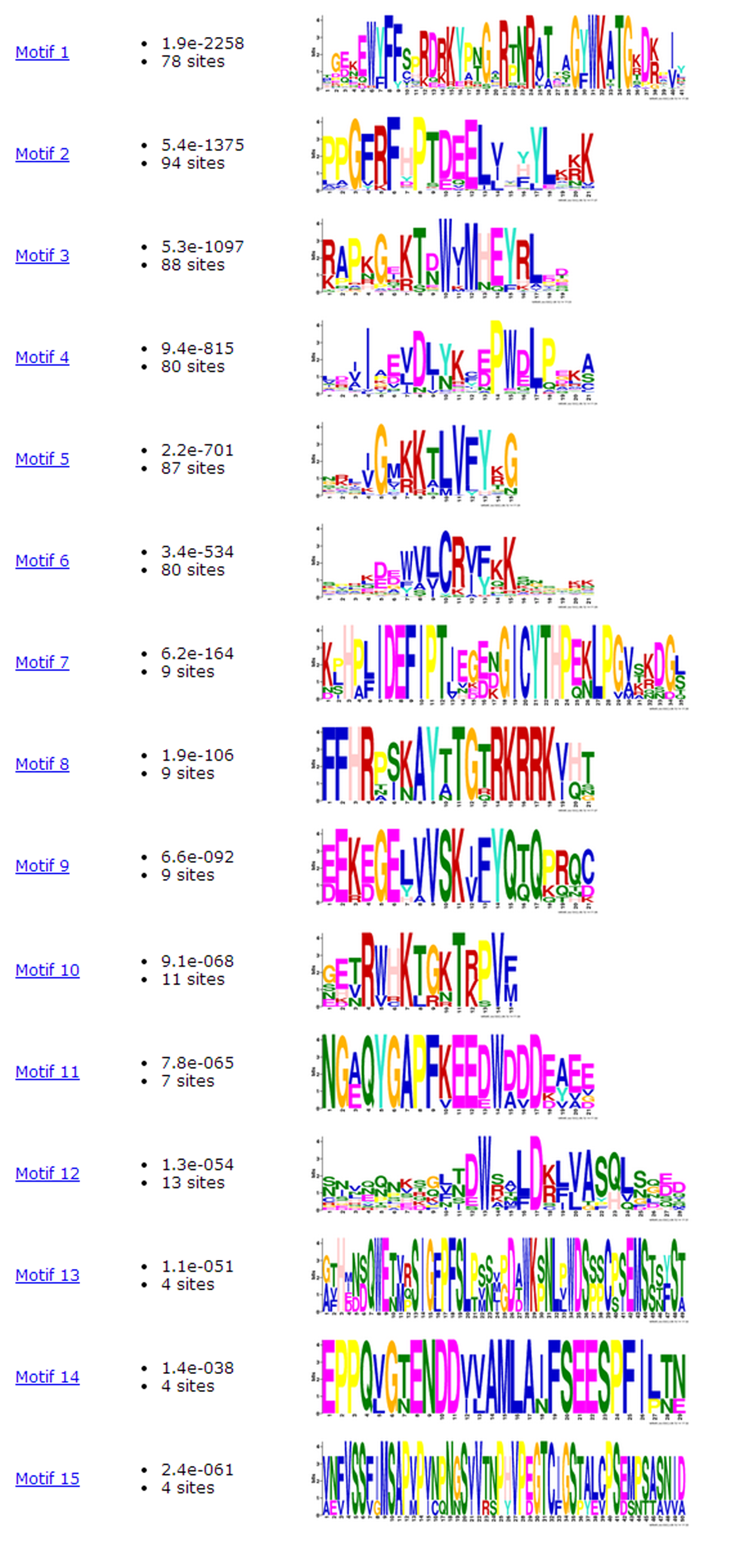

Supplement: S1 Fig — (TIF) [file pone.0136993.s009.tif]
